# Pre-service Teachers Learning to Teach English as a Foreign Language to Preschool Learners in Macau: A Longitudinal Study

**DOI:** 10.3389/fpsyg.2021.720660

**Published:** 2021-08-10

**Authors:** Barry Lee Reynolds, Sylvia Liu, Xuan Van Ha, Xiaofang Zhang, Chen Ding

**Affiliations:** ^1^Faculty of Education, University of Macau, Macao, China; ^2^Centre for Cognitive and Brain Sciences, University of Macau, Macao, China; ^3^Department of Foreign Languages, Ha Tinh University, Ha Tinh, Vietnam

**Keywords:** English as a foreign language, pre-service teachers, pre-primary education, preschool education, teacher beliefs, teacher learning, teacher education

## Abstract

Language teacher beliefs have received increasing research attention for the past few decades. However, little is known about the beliefs of pre-service teachers in the pre-primary English as a foreign language (EFL) education context. This qualitative case study extends this line of inquiry by investigating the trajectory of student teachers' beliefs about teaching English to pre-primary learners in Macau within a teacher education course. The participants included 60 pre-service teachers taking an *English Language Activities* course in their third year of a 4-year Bachelor of pre-primary education program. The data comprised written reflections collected at three points in time during the 16-week course: at the beginning of the course, mid-way through the course, and at the end of the course. The findings showed five broad themes, constituted from 15 subthemes, regarding (1) learners and learning, (2) teaching, (3) subject, (4) self, and (5) learning to teach. The major themes have been documented in the literature, but several subthemes were identified for the first time in the context of pre-primary EFL teacher education. More importantly, the findings revealed that some of the subthemes were newly shaped and several subthemes were reshaped as a consequence of taking the course. The findings were interpreted in relation to the content of the course, the experiential learning opportunities, the pre-service teachers' prior experiences of language learning and teaching, and the local language teaching and learning context. Implications for pre-service teacher education programs are discussed.

## Introduction

Teachers' beliefs are shaped by experiences. Critical times in teachers' lives, such as when they receive teacher education, are especially important in shaping, solidifying, and changing their pedagogical beliefs. The beliefs held by teachers have been shown to influence their classroom teaching (Borg, 2003; Ha and Murray, 2020), which subsequently influences their students' learning. Therefore, it is important to understand whether and how teacher education can change pre-service teacher beliefs that will lead to effective learning outcomes. As the English language education students receive from pre-primary teachers during early childhood education (ECE) could have lasting effects well into adulthood, it is of paramount importance to understand how teacher education in pre-primary education programs can influence the beliefs pre-service pre-primary teachers have about English teaching and learning. This is especially important in teacher education contexts such as the Macau Special Administrative Region (SAR) of China, in which the amount of language teacher education that pre-service pre-primary teachers receive is limited. Thus, a case study was undertaken to track the beliefs of a group of pre-service pre-primary teachers from the Macau SAR that were receiving English teacher education and training.

## Literature Review

### Teacher Beliefs

Beliefs are defined as “propositions individuals consider to be true and which are often tacit, have a strong evaluative and affective component, provide a basis for action, and are resistant to change” (Borg, 2011, pp. 370–371). Teacher beliefs have been shown to influence their classroom behaviors, and understanding teachers' beliefs is important because it helps to understand teachers' teaching practices (Borg, 2003; Ha and Nguyen, 2021). In the context of teacher education, beliefs are considered to be an essential element in teacher learning (Borg, 2011; Ha and Murray, 2021). Various studies have investigated teacher beliefs about teaching, learning and learners, subjects, and self as a teacher (Calderhead, 1996; Borg, 2003). This body of literature has shown that teacher education is more likely to influence teachers' practices than any other force.

A considerable number of studies have indicated that teacher education impacts teachers' beliefs, professional development, and subsequent teaching practices (Zheng, 2009; Tang et al., 2014). Pre-service teachers may hold biased teaching beliefs at the beginning of their training stages (Clark-Goff and Eslami, 2016); for example, some pre-service teachers ideate that “teaching and learning” are “telling and memorizing” (Zheng, 2009). Therefore, pre-service teachers need somehow to eliminate their pre-existing misconceptions about teaching and learning; doing so will result in fewer difficulties being encountered during actual future teaching (Zheng, 2009; McLean et al., 2013). Fortunately, when compared to in-service teacher beliefs, pre-service teacher beliefs are more changeable. Because student teachers have not had time to form certain teaching patterns in the beginning stage of becoming teachers, if these unfounded beliefs are changed, their future teaching practice and students' learning can be positively affected (Zheng, 2009; Voss and Kunter, 2019). For instance, Akaba et al. (2020) examined pre-service teachers' beliefs concerning school policy adjustments, pinpointing that “individualized, localized support” and “coherent communication” (p. 13) between policy makers and pre-service teachers can largely affect pre-service teachers' teaching experiences as well as improving their teaching quality. Their findings showed that pre-service teachers long for more pedagogical autonomy during the policy decision-making process and teaching practice. Additionally, same as in-service teachers, pre-service teachers' future professional and personal development can be swayed by their beliefs (Zheng, 2009; Lin, 2013; Yuan and Zhang, 2017). Specifically, pre-service teachers who consider teaching as a job and not a “life-long learning process” (Zheng, 2009, p. 77) would not realize the importance of professional development.

### English as a Foreign Language Teacher Beliefs

In the fields of both general education and language education, the significance of teacher beliefs has been widely acknowledged. In addition to the English subject, teacher beliefs about other knowledge domains have also been investigated in the existing literature (e.g., Atabek and Burak, 2020; Yuan et al., 2020). However, differing from other subject teachers, previous studies have shown that English language teachers usually hold mixed motivations and beliefs, including altruistic (e.g., desire to teach children and influence their future development), intrinsic (e.g., interests in English language or in teaching itself), and extrinsic (e.g., salary, social support, and working environment) (Yuan and Zhang, 2017). Zheng (2009) pointed out that instead of spending time on learning pedagogical knowledge within their education programs, pre-service English language teachers prefer utilizing the majority of their time to enhance their English proficiency. Thus, insufficient pedagogical knowledge will certainly affect their teaching beliefs as well as teaching practice. Therefore, different pedagogical methods are necessary to be introduced to English teachers so that they can adapt and apply these approaches to their unique teaching contexts, adjust the existing teaching approaches based on their own teaching experience, and then create more flexible and student-oriented English learning methods (Lin, 2013).

Pre-service teacher beliefs have been investigated at all levels of education, including college instructors (Aksoy, 2017), high school teachers (Cheung and Hennebry-Leung, 2020), primary school teachers (Atabek and Burak, 2020), and pre-primary school teachers (Munez et al., 2017; Alacam and Olgan, 2019). These studies have generally found that novice teachers often feel stressed when receiving teacher training and feel disenchanted or even experience emotional exhaustion during the actual teaching practice (Voss and Kunter, 2019).

Regarding pre-service pre-primary English teachers, educational researchers have focused on exploring the relationship between teacher beliefs and teaching practice (Cheung and Hennebry-Leung, 2020; Albaiz and Ernest, 2021), parent involvement (Alacam and Olgan, 2019) and professional development (Brown, 2016; Munez et al., 2017). The results of these studies have shown pre-service pre-primary teachers' interactions with students, schools, parents, and professional development activities all impacted the formation of teacher beliefs. For example, Alacam and Olgan (2019) found that pre-service pre-primary teachers generally were highly confident in organizing parent involvement activities while having low skills in handling the relationships with parents. Albaiz and Ernest (2021) recommended teaching practices could include parent involvement and community collaboration. Parent involvement activities within teacher education programs could help pre-service teachers learn how to form and maintain relationships with parents. Munez et al. (2017) also found that pre-service pre-primary teachers possessed higher levels of self-efficacy beliefs when they engaged in collaborative professional development activities, which indicated that such activities (e.g., peer-observation, teacher networks, professional learning communities) should be highly valued by pre-service pre-primary teachers.

### Change in Teacher Beliefs

Contradicting results have been found in the studies investigating changes in teacher beliefs. While some researchers found pre-primary teacher beliefs to be inflexible and resistant to change (e.g., Calderhead, 1991; Johnson, 1994; Peacock, 2001; Urmston, 2003), others found that reflective practice and education courses provide support in reshaping teacher beliefs (e.g., Cabaroglu and Roberts, 2000; MacDonald et al., 2001; Bekleyen, 2011). For example, Calderhead (1991) found that the British pre-service teachers involved in his study had fixed images of themselves as teachers. These images were mostly drawn from the memories of the teachers from their youth. Additionally, their beliefs about teaching remained predominantly constant during the teacher education course and practicum. Johnson (1994) investigated the beliefs of pre-service English as a second language (ESL) teachers who had no previous teaching experience and were in their first year of a Masters of Arts program, majoring in teaching ESL. Johnson's findings showed that the most influential factor on pre-service teachers' beliefs and teaching practice was their prior experience as second language learners and that these beliefs were resistant to change. On the other hand, Yuan et al. (2020) investigated the efficiency of video-based reflective practice with six pre-service primary teachers. They found that the teachers developed “critical thinking and reflective abilities” (p. 16) through engaging with their peers in a reflective learning environment.

It has been suggested that to remedy this resistance, teacher education courses should create opportunities that allow such beliefs to be explored by the pre-service teachers (Altan, 2012). Teacher education courses should allow pre-service teachers to challenge their preconceptions about teaching and encourage more reflective practices throughout the course (Calderhead, 1991; Cabaroglu and Roberts, 2000; Zheng, 2009).

Teacher education can improve pre-service teachers' knowledge about the subject matter, learners, learning, and teaching approaches, and it can shape or reshape their pedagogical beliefs (Chan, 2016). In general, teacher education seems to prepare pre-service teachers well for the transition into the period of actual teaching, even though some studies have indicated that a gap may still exist between teacher education theory and teacher education practicums (Korthagen et al., 2006). For example, after experiencing teacher education, pre-service teachers were shown to be inclined to hold more “transmissive beliefs” instead of their previous “constructivist beliefs” (Voss and Kunter, 2019, p. 3). Transmissive beliefs refer to the teacher-oriented knowledge transfer from teachers to students, while constructivist beliefs stress students' learning process of conceptual understanding. On the one hand, although very few studies have investigated whether the changes in teacher beliefs caused by teacher education are transient or may have a long-lasting effect (Levin, 2015; Voss and Kunter, 2019), the results of Voss and Kunter's (2019) study indicated that pre-service teacher beliefs are relatively unchangeable. On the other hand, several studies have shown that pre-service teachers' beliefs can be changed by certain forces, for instance, pre-service teachers' previous learning experience (Sinclair, 2008), expectations of the teaching results (Sinclair et al., 2006), the perception of their own teaching capabilities (Siwatu, 2007), social resources and guidance that support pre-service teachers' professional learning (Tang et al., 2014), and teacher training programs (Tang et al., 2014; Yuan and Zhang, 2017). The results of these studies have indicated that pre-service teachers' beliefs are strongly influenced by their perception of their own teaching capabilities as well as their outcome expectations (Sinclair et al., 2006; Siwatu, 2007). In particular, Yuan and Zhang (2017) explored these personal and contextual factors by looking into how pre-service teachers' beliefs, especially their motivations, were shaped by their “self-efficacy, outcome expectations, professional autonomy, and social support” (p. 142). They found that student teachers were able to increase their knowledge about language teaching and become familiar with their future work contexts by engaging in their teacher education program (Yuan and Zhang, 2017). Additionally, teacher education not only can reinforce teacher efficacy (Zimmerman, 2008) but is also able to form optimistic expectations about teachers' professional learning (Siwatu, 2007; Yuan and Zhang, 2017). Therefore, when implementing teacher education programs, policy makers and curriculum designers need to conceive a coherent program that considers pre-service teachers' personal interests, professional needs, and social support (Yuan and Zhang, 2017).

All in all, teacher education provides pre-service teachers with opportunities to go through an authentic teaching practice as well as develop their professional capabilities, which may result in a change in their beliefs (Sinclair, 2008; Yuan and Zhang, 2017). However, little research has focused on changes in teacher beliefs caused by teacher education in the language teaching context of Macau. Therefore, this study aims to investigate whether and how pre-service teacher education in Macau may change pre-primary English teacher beliefs about the teaching of English to very young learners. The following research question was proposed to guide this investigation:

To what extent and how do pre-service pre-primary teacher beliefs about teaching English as a foreign language to young learners in Macau change throughout a teacher education course?

## Methods

A qualitative case study design was used to gather data in order to allow for a “close and extended analysis of the particular” (Hood, 2009, p. 66). In the following subsections, the participants, learning context, and data collection are described. The research methodology was reviewed by the University of Macau ethics review board and approved under reference number SSHRE19-APP071-FED.

### Participants and Learning Context

Sixty year-three Bachelor of Pre-primary Education students from one university in the Macau SAR of China were recruited as participants. The Bachelor of Education in pre-primary education is a 4-year 121-credit program in which the majority of courses use Chinese as the language of instruction. These students were females between 20 and 21 years old with a threshold or intermediate English language proficiency (B1 on the Common European Framework of Reference for Languages). In addition to major-related pre-primary education courses, these students were required to complete compulsory and general education courses, elective courses, and a 1-year teaching practicum in their fourth year of studies. Only one compulsory course within the program focused on providing these students with pedagogical knowledge related to teaching English as a foreign language (EFL); the students participated in the study during their third year of studies while enrolled in this course. No other courses in the university were available to the participants that focused on teaching English to preschool students. Upon graduation, the graduates of the program are certified to teach in kindergartens within the Macau SAR.

### Teacher Education Course

The participants were enrolled in *English Language Activities*, a 16-week course that aimed at preparing pre-service teachers to teach EFL to pre-primary pupils. The course emphasized the use of various language activities to teach EFL (e.g., games, songs, nursery rhymes, role plays, storytelling). In addition, students enrolled in the course learned to design, adopt, and adapt different teaching materials and teaching aids when planning English lessons. The students also gained a better understanding of various language teaching approaches, underlying theories, and lesson planning. Course topics included issues on children learning a foreign language; creative classrooms for very young language learners and child-centered lessons; lesson planning for young language learners; children learning language through tasks and activities; young learner assessment; children learning through stories; creating, adapting, and evaluating activities for young language learners; games and songs for young language learners; children learning words; and language choice and language learning.

The majority of class sessions were devoted to lecturing, class discussion of assigned readings, preparation and execution of microteaching practice, and analysis of and class discussion about the pre-service teachers' peer microteaching. Students were expected to read and review assigned course readings before class. Students also wrote 10 min lesson plans that included information on the target students, rationale, goals, objectives, materials, assessment, and procedures. They should have taken notes during class and reflected on these notes to articulate their beliefs about teaching EFL. The course instructor provided individual written feedback on the microteaching lesson plans and oral feedback in class during the analysis of the peer microteaching sessions. Students were further assessed through (1) a participation score related to their contribution to class discussions about the course readings, feedback provided to their classmates' peer microteaching, and attendance; (2) a written lesson plan; (3) in-class peer microteaching of the lesson plan; and (4) a final exam that required the students to watch a video of a lesson taught to very young EFL learners and then provide written constructive feedback (i.e., praise and criticism) of the lesson and then to reflect on their own teaching practice by writing a teaching philosophy statement concerning the teaching of English to very young language learners.

### Data Collection

Data collection occurred at three points in time across one academic semester in the course described above. Data were collected at the start of the course (week 1), mid-way through the course (week 9), and at the end of the course (week 16). The data was collected in the form of reflection reports written by the students containing their beliefs about teaching and learning EFL in the Macau SAR kindergarten context. The same prompt (see Appendix A) was provided by the researchers to the students at all three times, and the students were given 2 h to write their reflections in either English or Chinese. Sixty reflection reports were collected at each of the three times. A total of 12,282 word tokens (*M*_tokens_ = 204.7; *SD*_tokens_ = 76.24) was written by the participants at the beginning of the course (Time 1), 14,605 word tokens (*M*_tokens_ = 243.42; *SD*_tokens_ = 108.03) was written by the participants mid-way through the course (Time 2), and 21,431 word tokens (*M*_tokens_ = 357.18; *SD*_tokens_ = 102.50) was written by the participants at the end of the course (Time 3). The majority of students wrote their reflections in English at the beginning of the course (78.33%, *N* = 47) while 100% of the participants wrote their reflections in English mid-way through the course and at the end of the course. The reflection reports written in Chinese were translated into English by the second author prior to analysis.

### Data Analysis

The data were analyzed with the support of NVivo 12, a qualitative content analysis (QCA) software, as it could increase this case study's reliability, rigor, and trustworthiness (Leavy, 2014). The QCA included five steps (Schreier, 2012). First, all 180 reflection reports were anonymized and read three times by the researchers and coders to obtain a holistic sense of the data. During this step, some content areas of the reflection reports were highlighted to assist with a general understanding of the data. This step allowed the researchers to become familiar with the rich data and to generate preliminary categories. For example, the following comment was highlighted as “Beliefs about Self-evaluation.”

In order to make my teaching efficient, I will regularly evaluate myself as well. I will record my lesson and write reflections on it from time to time. I will also invite my colleagues to give me some advice on my teaching. Besides[,] I will also watch videos of other teachers['] class[es] to compare with mine.

**Figure 1:**
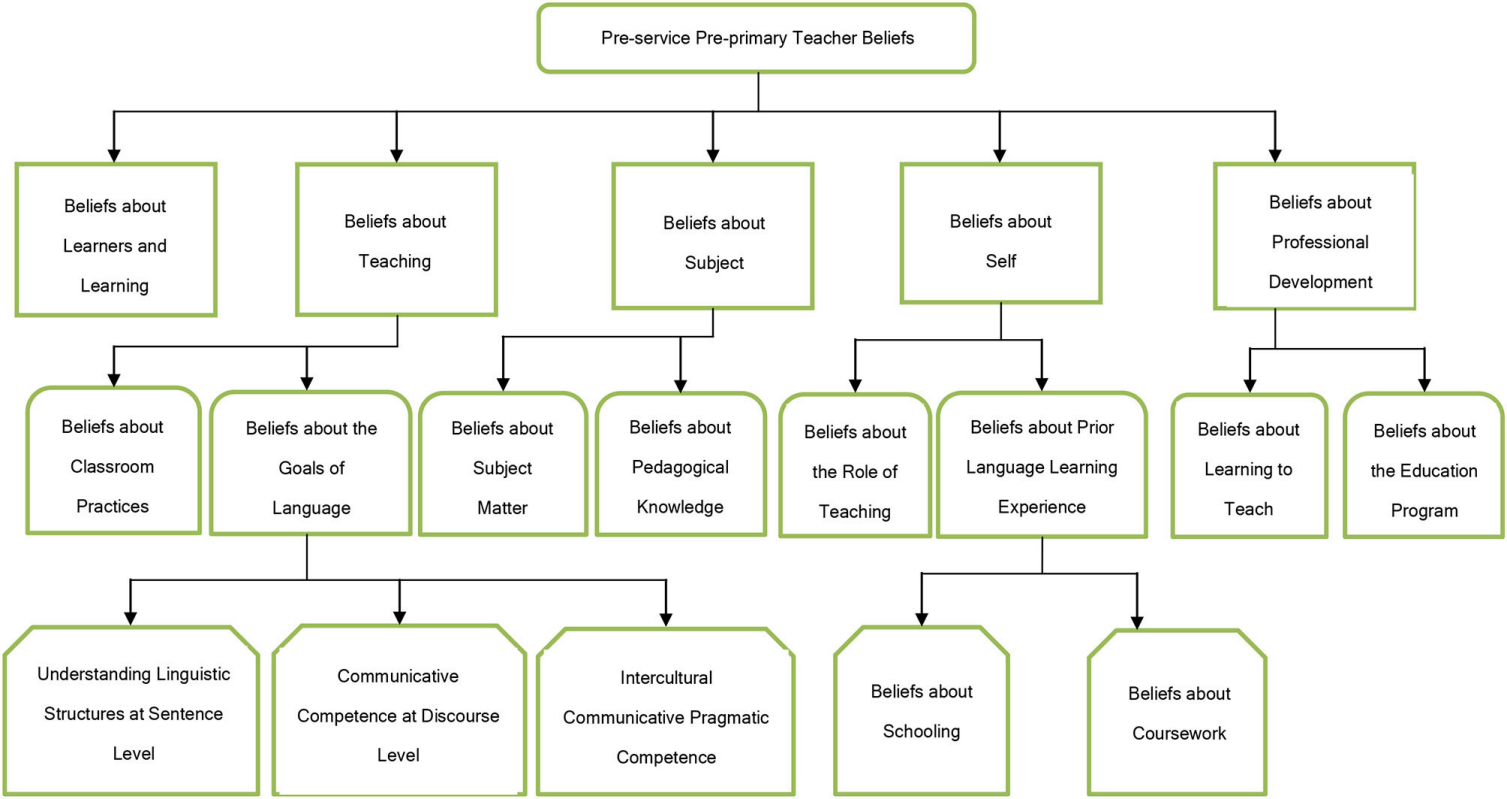
The concept-driven coding frame.

**Figure 2:**
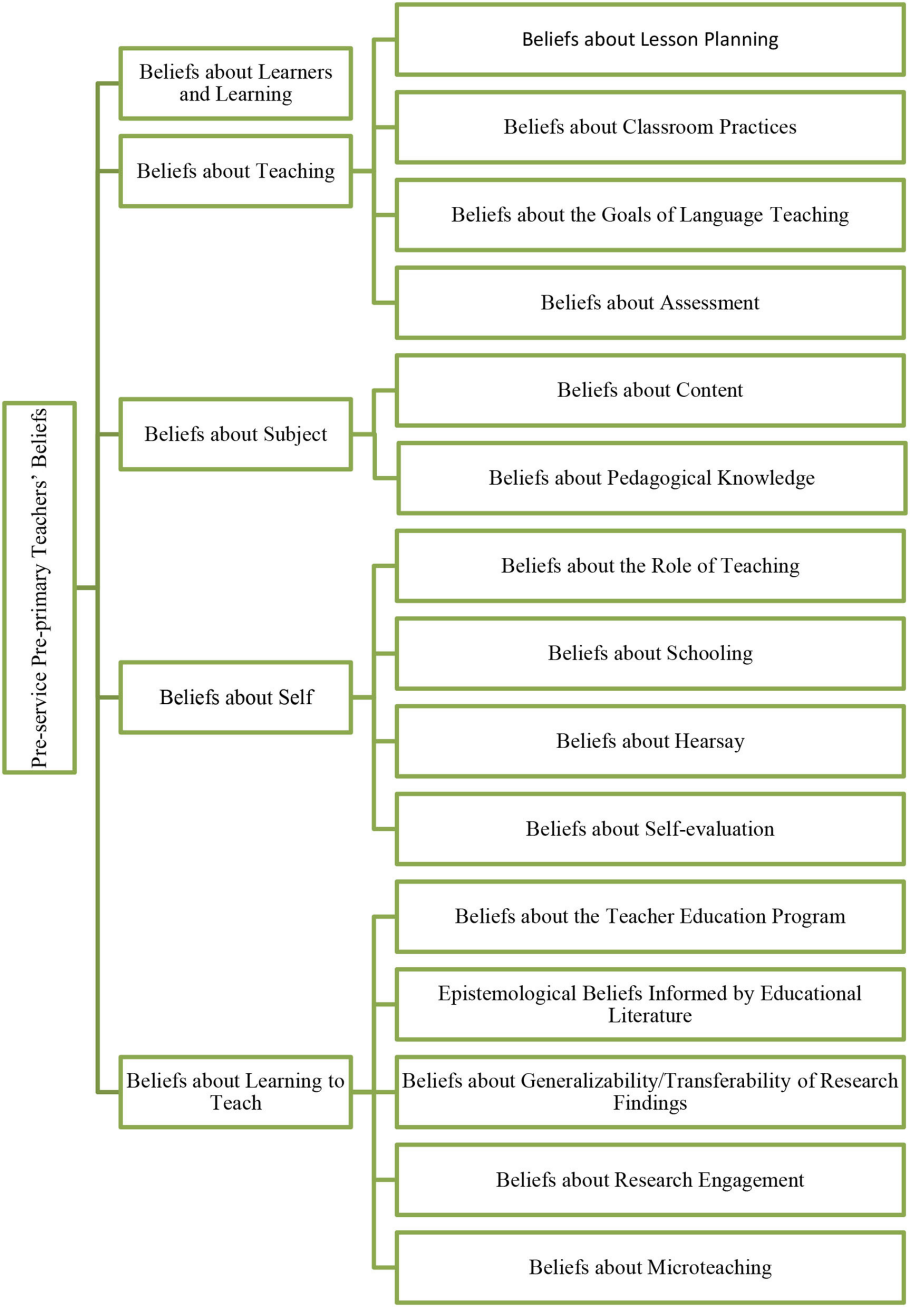
Pre-service pre-primary teachers' beliefs.

Second, generating (sub)categories from both the data and the literature, the researchers built a concrete coding frame to organize the (sub)categories by name, operationalization, and example. This two-phase process is illustrated in Figures 1, 2. In the first phase, relevant literature was reviewed to develop a preliminary coding frame. With related literature on hand, five main categories and 13 subcategories were identified (Wall, 2016; Gilakjani and Sabouri, 2017; Vosniadou et al., 2020) (see Figure 1). In the second phase, the two coders used this coding frame to conduct a pilot coding with 40 randomly selected reflection reports. During the pilot coding, the two coders use *in vivo* coding to create the (sub)categories. The initial *in vivo* codes taken directly from the participants' reflections were replaced with a concise label. For example, the sampling *in vivo* code from participant #27 was “The microteaching is a good practice for us to try and share ideas on planning activities for the kindergarteners. It enables us to learn from one another and inspires some of our new thoughts,” which was collapsed to “technique in teacher training that uses short, specific episodes.” This quote was later coded under the data-driven subcategory “beliefs about microteaching” under the main category “learning to teach.” In this way, the concept-driven preliminary coding frame was refined to better reflect the data (see Figure 2).

Third, after completing the refined coding frame, the two coders coded the 180 reflection reports independently by using NVivo 12. Chunks of statements that captured participants' beliefs were placed in appropriate (sub)categories during this step. As the two coders conducted this step individually, a set of consistent and valid coding procedures were followed.

Fourth, the second cycle of coding was conducted by the two coders. This step confirmed the first coding and aimed to ensure the (sub)categories were sufficiently distinct from each other. Lastly, all the researchers engaged in a final read of the coding results. Co-occurrences of beliefs were then carefully examined among three different periods. The changes of the beliefs were highlighted and interpreted for further thematic description.

To ensure valid and reliable findings, the two coders used a two-cycle procedure for data coding. This procedure was carried out independently by two coders with different educational backgrounds (i.e., Early Childhood Education and English Education). After the first round of coding, any discrepancies were discussed by the coders. The second round of coding was then completed to ensure consistency. The percentage of agreement was above 0.99 which indicated excellent inter-rater reliability (Miles and Huberman, 1994). Moreover, the coding frame was examined by an external expert familiar with both QCA methods and teachers' beliefs; this expert evaluation ensured the validity of the QCA (Schreier, 2012).

## Results

Five beliefs themes with 15 subthemes emerged from the QCA of the pre-service pre-primary teachers' reflection reports. Table 1 presents an overview of the emerging (sub)themes and the percentage of the participants who held each belief (sub)theme at each time point.

**Table 1:** Pre-service pre-primary teachers' beliefs.

**Beliefs Theme**	**Time 1** **Number of Participants that Held the Belief**	**Time 1** **Proportion of Participants that Held the Belief (%)**	**Time 2** **Number of Participants that Held the Belief**	**Time 2** **Proportion of Participants that Held the Belief (%)**	**Time 3** **Number of Participants that Held the Belief**	**Time 3** **Proportion of Participants that Held the Belief (%)**
Beliefs about learners and learning	53	88.33	22	36.67	45	75.00
Beliefs about teaching	33	55.00	34	56.67	31	51.67
Beliefs about lesson planning	0	0.00	7	11.67	2	3.33
Beliefs about classroom practices	55	91.67	40	66.67	38	63.33
Beliefs about the goals of language teaching	13	21.67	7	11.67	32	53.33
Beliefs about assessment	0	0.00	2	3.33	46	76.67
Belief about subject	12	20.00	6	10.00	9	15.00
Belief about content	15	25.00	10	16.67	8	13.33
Beliefs about pedagogical knowledge	41	68.33	42	70.00	60	100.00
Beliefs about self	5	8.33	17	28.33	0	0.00
Beliefs about the role of teaching	14	23.33	15	25.00	17	28.33
Beliefs about schooling	12	20.00	3	5.00	11	18.33
Beliefs about hearsay	8	13.33	2	3.33	1	1.67
Beliefs about self-evaluation	0	0.00	0	0.00	34	56.67
Beliefs about learning to teach	4	6.67	21	35.00	28	46.67
Beliefs about the teacher education program	3	5.00	2	3.33	2	3.33
Epistemological beliefs informed by educational literature	0	0.00	0	0.00	40	66.67
Beliefs about generalizability/transferability of research findings	0	0.00	0	0.00	10	16.67
Beliefs about research engagement	0	0.00	0	0.00	1	1.67
Beliefs about microteaching	0	0.00	16	26.67	0	0.00

### Learners and Learning

This theme encapsulates participants' beliefs about young children whose first language is not English (Harris and Hodges, 1995). Nurturing young children's interests were mentioned by the majority of participants as discovering a “way” that truly motivates learning. For example, participant #20 said, “At the pre-primary level, kids should learn [a] foreign language in a[n] interesting way.” These “interesting way[s]” included both EFL child-centered learning activities (e.g., Participant # 33) and environments (e.g., Participant # 46). In terms of the “learning activities,” participants believed games and stories supported EFL learning and a relaxed environment nurtures EFL learning.

Most of the participants' beliefs about EFL learners and learning changed while enrolled in the course. Fifty-three participants (88.33%) mentioned their beliefs about EFL learners and EFL learning at the start of the course, 22 (36.67%) mid-way through the course, and 45 (75.00%) at the end. However, only five participants adopted new beliefs about EFL learning and EFL learners. Gradually, they placed importance on nurturing young children's interests, as participant #18 put it, “children are willing to put more effort in things that they are interested in.”

### Teaching

Beliefs about teaching are referred to as teachers' preferred methods and techniques of teaching (Teo et al., 2008). Fifty-four participants (90%) believed teaching involved not only classroom practices but goals, lesson planning, and assessment. For example, participants #5, #34, and #56 wrote about effective classroom practices being the key to keeping very young children interested, engaged, and more likely to follow teachers' instructions. Eight participants (i.e., #2, #3, #6, #7, #20, #27, #42, and #59) believed well-designed lesson plans can facilitate teaching. Specifically, participant #33 believed that teaching should encourage children's curiosity and changing her lesson plan could result in disinterested students becoming interested.

Thirty-three participants (55%) mentioned their beliefs about teaching at the start of the course, 34 (55.67%) mid-way through, and 31 (51.67%) at the end; six participants (10%) did not state any beliefs related to this theme throughout the course. Most of the participants reflected on this belief at the second data collection point, right after they completed their in-class peer microteaching. In addition, 18 of the 34 participants that reflected on their beliefs about teaching did so for the first time mid-way through the course.

#### Lesson Planning

Nine participants (15%) mentioned their beliefs about lesson planning or the “objectives, procedures, and materials for…learning activit[ies]” (Harris and Hodges, 1995, p. 137). In most cases, a lesson plan was seen as a vital component of teaching which kept the pre-service pre-primary teachers prepared and on schedule. In particular, participant #2 believed “[a] precise plan for every English class” including the description of the steps can remind her of all the steps she needs to take in class.

Seven participants (11.67%) reflected on lesson planning for the first time mid-way through the course and two participants (3.33%) at the end. No participants mentioned beliefs about lesson planning at the start of the course. Fifty-one participants (85%) did not state any beliefs related to lesson planning. This result cannot necessarily be interpreted as the participants having limited beliefs about lesson planning. Some participants provided concrete examples of how they might carry out particular classroom practices. While not specifically referring to the organization of activities as lesson planning, such reflections were coded under other (sub)themes that did not require subjective interpretation.

#### Classroom Practices

Beliefs about classroom practices are defined as “[c]lassroom practice, as a process, involv[ing] multiple agents and their interactions within the classroom as a system. The process can be manifested in diverse formats and structures, and its effectiveness can be influenced by numerous factors both internal and external to the classroom” (Li and Oliveira, 2015, p. 489). Beliefs about classroom practices were held by 59 participants (98.33%). Participants reported that the class atmosphere should be positive and filled with early childhood classroom activities that actively engage very young children in cooperative English learning activities. Furthermore, they believed classroom assessments should help teachers identify individual learning needs.

Fifty-five participants (91.67%) mentioned beliefs about classroom practices at the start of the course, 40 (66.67%) mid-way through, and 38 (63.33%) at the end. Twenty-four participants (40.00%) held these beliefs throughout the course, although with some reformation of the beliefs. For example, at the start of the course, participant #1 mentioned the use of total physical response (TPR) for teaching learners: “I may tell a story with drama or exaggerate act[ion] and expression; therefore[, students] may easily understand what the story is about and they may [learn] some words in the story.” By the end of the course, she further elaborated on her use of TPR and how she would use assessment to facilitate learning through TPR. Only one participant did not state any beliefs about classroom practices and only one participant changed her beliefs about classroom practices by the end of the course; most of the other participants changed their beliefs in a more moderate way by elaborating on a previously mentioned belief earlier in the course.

#### Goals of Language Teaching

Thirty-seven participants (61.67%) mentioned their beliefs about the goals of language teaching which is defined as beliefs about the “creat[ion of] skillful L2 users with all their extra attributes, not shadows of native speakers” (Cook, 2013, p. 51). In setting goals for teaching very young learners, the participants held beliefs about age-appropriate instructions (e.g., Participant #16), language stimulation activities (e.g., Participant #42), a joyful classroom atmosphere (e.g., Participant #4), and encouraging happy and fearless engagement in class (e.g., Participant #18).

There were 13 participants (21.67%) who mentioned beliefs about the goals of language teaching at the start of the course, seven (11.67%) mid-way through, and 32 (53.33%) at the end. Twenty-three participants (38.33%) did not state any beliefs related to this subtheme, and most participants reflected on new beliefs about the goals of language teaching after they completed the entire course.

#### Assessment

Forty-six participants (76.67%) mentioned beliefs regarding “the act or process of gathering data in order to better understand the strengths and weaknesses of student learning, as by observation, testing, interviews” and other teaching techniques (Harris and Hodges, 1995, p. 12). Specifically, these participants believed that picture matching games are age-appropriate assessment tools. For example, participant #35 explained that “For assessment, [if the] children still remember [what] I taught, I may also let them [complete a] picture matching [activity] if the time allow[s].” Other participants (e.g., participant #54) also referred to various ways to match colors or spoken words to pictures or cards to assess children's word knowledge.

Two participants (3.33%) mentioned beliefs about assessment mid-way through the course, and 46 (76.67%) participants mentioned these beliefs at the end of the course. This resulted in 44 participants reflecting on beliefs about assessment at the end of the course that did not do so at any earlier points in the course. Furthermore, none of the participants mentioned any beliefs about assessment at the start of the course. Apart from this, 14 participants (23.33%) did not state any beliefs about assessment at any of the data collection points. The participants had not previously received any training on how to engage very young learners in language assessment. However, the course offered specific training in appropriate test design and formatting (see Hughes, 2013), likely to have led to the participants' beliefs regarding assessment to become more salient.

### Subject

Beliefs about the subject are teachers' beliefs toward “[a]n area of learning and study; discipline” (Harris and Hodges, 1995, p. 246). Beliefs about the subject were held by 21 participants (35%), the majority of whom believed English is an important subject for young children. For example, many participants claimed English is an important global language that is a necessary tool for communication (e.g., participant #24).

Twelve participants (20.00%) mentioned beliefs about subject at the start of the course, six (10.00%) mentioned beliefs about subject mid-way through the course, and nine participants (15.00%) mentioned beliefs about subject after they completed the course. Furthermore, four participants reflected on new beliefs mid-way through the course, and six participants expressed beliefs about subject for the first time at the end of the course. Only participant #58 (1.67%) reflected on beliefs about the subject at all three data collection time points. She expanded her beliefs about the subject from a child-focused level (i.e., “English will play an important role during [very young learners'] growth.”) to a context-focused level (i.e., “Nowadays, English is one of the important language[s] around us.”) until finally an international-focused level (i.e., “English is a widely used international language.”) In addition, 39 participants (65%) did not state any beliefs about the subject at any data collection point.

#### Content

Twenty-four participants (40%) mentioned their beliefs about “[k]nowledge of the subject and its organizing structures” (Ball et al., 2008, p. 2). Participants believed very young learners can learn best when content is age-appropriate (e.g., participant #4) as ensuring this will foster communicative competence (e.g., participant #8).

Fifteen participants (25%) mentioned beliefs about content at the start of the course, 10 (16.67%) mid-way through, and eight (13.33%) at the end. In addition, five participants reported new beliefs about content mid-way through the course, and four participants reported new beliefs about content after they completed the course. Only two participants (3.33%) held beliefs about content throughout the course and 36 participants (60%) did not state any beliefs about content. As very young learner EFL classroom content is usually the same as other subjects but taught and learned in English (Reilly and Ward, 1997), the participants likely started to perceive their previous early childhood teacher education as transferrable to the very young EFL learner classroom.

#### Pedagogical Knowledge

All participants (100%) held beliefs about “[p]edagogy as the art or science…of being a teacher, involving methods and techniques of teaching predicated on two conceptions of pedagogy: the liberal, emphasizing the autonomy of the child; and the conservative, emphasizing the authority of the teacher” (Farquhar and White, 2014, p. 822). The participants believed ECE and EFL pedagogies should help in determining “[H]ow I will teach English as a foreign language at the pre-primary level” (e.g., participant #7). Their beliefs about pedagogical knowledge revolved around teaching methods that enabled successful learning while offering opportunities for children to use English to foster communication skills and learning interests within a learner-centered classroom environment. The participants also believed that pedagogical knowledge should be fueled by interactive processes. For example, participants #15, #35, and #46 believed that pedagogical knowledge could be enhanced through daily exploration and interactions with young learners.

In addition, some participants believed certain pedagogies can better facilitate their future teaching. Firstly, participants believed that the cultivation of very young EFL learners' interest in learning English plays a crucial role in how successful their teaching will be. For instance, participant #37 believed EFL teachers should “tak[e] the interest of young students as the center [of their teaching].” Participants believed they should pay attention to what activities the very young EFL learners are interested in to foster “interest-centered” classes (e.g., participant #3); these participants believed pre-primary teachers should first observe and then scaffold learning through various game play. Secondly, “games” were believed to be a highly important pedagogical component aimed at promoting very young EFL learners' language abilities. The participants believed “game pedagogy” can increase learners' motivation; participant #41, for example, wrote that “learning [English] through games and using games [when teaching] can increase children's motivation and interest in learning.” Thirdly, participants believed that the implementation of ECE pedagogies in combination with EFL pedagogies and a child-centered classroom is the best combination for enhancing their teaching of English. This eclectic method can promote “listening and speaking” (e.g., participant #20) and increase the amount of active engagement by learners (e.g., participant #1).

The participants built up these beliefs about pedagogical knowledge throughout the course. More specifically, 13 different types of pedagogies were mentioned before the course began and 30 different types of pedagogies were mentioned at the end of the course. Furthermore, most of the participants changed their beliefs about pedagogical knowledge as a response to participation in the course. There were 41 participants (68.33%) who mentioned various pedagogical beliefs at the start of the course, 42 (70%) mid-way through, and 60 (100%) at the end. In addition, nine participants reported new beliefs mid-way through the course, and 10 participants that expressed no beliefs about pedagogical knowledge at the start or mid-way through the course did so by the end. These participants' beliefs were basically built up through a gradual gathering of their reflections on the pedagogies that the course lecturer introduced to them. Participant #62 is a good example as her reflection reports indicated a gradual realization of the importance of integrating different pedagogies to nurture young children's language learning.

### Self

“[T]eacher self-efficacy and teacher emotions can be important ways for…language teachers to enhance [their] overall quality” (Xu, 2012, p. 1400). Such beliefs about self as a teacher were mentioned by 20 participants (33.33%). While some participants reflected on their low confidence in using the English language in general and more specifically for teaching, others reflected on how they were confident in teaching English. For example, participant #34 claimed she was “a[n] encourage[ing] English teacher that [helps] children enjoy English class.”

Five participants (8.33%) mentioned beliefs about self at the start of the course, 17 (28.33%) mid-way through, and none at the end. In addition, 15 reported new beliefs mid-way through the course. Participants held more specific beliefs about self which were further broken down and discussed in the following sections: the role of teaching, schooling, hearsay, and self-evaluation.

#### The Role of Teaching

Thirty-four participants (56.67%) discussed “what teachers do in classrooms” (Keiler, 2018, p. 3). These beliefs included the different roles of a teacher. Generally, the participants expressed an understanding that their actions in the classroom are crucial in influencing their students' EFL learning. More specifically, participants #37, #44, and #57 described various coping mechanisms needed to fulfill their teaching roles. Fourteen participants (23.33%) mentioned beliefs about teaching at the start of the course, 15 (25%) mid-way through, and 17 (28.33%) at the end. In addition, 11 participants reported new beliefs mid-way through the course, and nine participants that did not express beliefs about the role of teaching at either the start or mid-way through the course did so at the end.

#### Schooling

Twenty-two participants (36.67%) reflected on “their own student experiences. [that] guide their interaction with and evaluation of ideas presented in course- and field-based experiences, causing them to accept, modify, or discount those ideas” (Schmidt, 2010, p. 132). Some of the participants' beliefs could be traced to critical moments in their own learning journeys. These included the participants' own English learning experiences and past professional learning experiences. Twelve participants (20%) mentioned these beliefs at the start of the course, three participants (5%) mid-way through, and 11 (18.33%) at the end. In addition, two participants mentioned new beliefs mid-way through the course, and eight that did not express any beliefs about schooling at the start or mid-way through the course did so at the end.

#### Hearsay

Ten participants (16.67%) described their beliefs that centered on “[w]hat is done or written, as well as to what is spoken” without evidence-based support (Falknor, 1940, p. 192). After hearing other people's English learning stories, the participants' beliefs changed. Eight participants (13.33%) mentioned such beliefs at the start of the course, two participants (3.33%) mid-way through, and one participant (1.67%) at the end. In addition, one participant reported new beliefs mid-way through the course, and only one participant that did not express any beliefs about hearsay at the start or mid-way through the course did so by the end. As the participants learned about research supported pedagogical practices through course lectures, readings, and in-class discussions, their beliefs about hearsay declined.

#### Self-Evaluation

Thirty-four participants (56.67%) referred to beliefs about “judging the quality of their work, based on evidence and explicit criteria, for the purpose of doing better work in the future” (Rolheiser and Ross, 2001, p. 1). The participants reflected on beliefs that served to remedy whatever inadequacies they believed they might have in their teaching. None of the participants mentioned beliefs about self-evaluation at the start or mid-way through the course. Thirty-four participants (56.67%) reported new beliefs after this course had been completed. They believed that self-evaluation could take on various forms such as written reflections on their teaching by considering what they have done or what they should do in the future. Furthermore, the participants perceived self-evaluation as an expression of commitment to their teaching competency development. This result was likely due to the reflective practice the teacher educator implemented in the course when the participants were required to reflect on their teaching of EFL in the Macau kindergarten classroom across three time points.

### Learning to Teach

Beliefs about learning to teach are teacher beliefs regarding “[k]nowing how to learn from classroom teaching experiences. It means planning these experiences in a way that affords learning and then reflecting on the outcomes in order to maximize the benefits that can be gained from the experiences” (Hiebert et al., 2003, p. 206). Thirty-six participants (60%) shared their beliefs about this theme. In general, they believed learning to teach is very important for pre-service teachers in Macau. Specifically, the participants believed pre-service teachers should learn how to formulate a teaching philosophy (e.g., Participant #2), choose suitable teaching methods (e.g., Participant #7), and administer age-appropriate assessments (e.g., Participant #48).

Four participants (6.67%) mentioned these beliefs at the beginning, 21 (35%) mid-way through, and 28 (46.67%) at the end of the course. In addition, 19 participants reported new beliefs mid-way through the course, and 13 participants that did not express any beliefs about learning to teach at the start or mid-way through the course did so at the end. More specific beliefs about learning to teach were categorized and discussed under the following sub-themes: beliefs about the teacher education program, epistemological beliefs informed by educational literature, beliefs about generalizability/transferability of research findings, beliefs about research engagement, and beliefs about microteaching.

#### Teacher Education Program

Seven participants (11.67%) described their beliefs regarding an “[e]ducation program which provides multiple opportunities for them to construct and reflect on learning to teach” (Kroll and Laboskey, 1996, p. 63). Generally, participants reported that the teacher education program prepared them to teach very young EFL learners in kindergartens through pedagogical knowledge that built up their confidence in teaching. They reflected on their conceptions of the teacher education program and that it had prepared them through instruction on different learning approaches (e.g., participant #25) and how to formulate teaching philosophies (e.g., participant #27). Participant #26 claimed the teacher education provided apprenticeship that “offers so [much] practical knowledge and really help[s] me a lot. Now I have more detailed [knowledge] to take into consideration.” Three participants (5%) mentioned such beliefs at the start of the course, two (3.33%) mid-way through, and two more (3.33%) at the end. The two participants that mentioned the beliefs about the teacher education program at the middle and end of the course did not mention such beliefs in previous reflections.

#### Epistemological Beliefs Informed by Educational Literature

Epistemological beliefs informed by educational literature are “conceptions of what counts as legitimate knowledge and how you know what you claim to know[. T]hese beliefs are particularly relevant for investigations of teachers' beliefs about educational research because research may be considered as a source of teaching knowledge” (Joram et al., 2020, p. 2). In general, the participants believed educational literature can inform learner-centered teaching (e.g., participant # 48), active teacher involvement (e.g., participant #32), and teaching through play (e.g., participant #4). None of the participants mentioned these beliefs at the start or mid-way through the course; however, 40 participants (66.67%) held these new beliefs at the end of the course. As this was the only course in the pre-primary education program that provided the participants with English language education literature, it is likely the course content that led to these changes in their beliefs.

#### Generalizability/Transferability of Research Findings

Beliefs about the generalizability/transferability of research findings refer to the processes of “applying the results of quantitative and qualitative research…to a population or context that differs from that of the original study” (Joram et al., 2020, p. 2). None of the participants mentioned such beliefs at the start or mid-way through the course; however, at the end of the course, 10 participants (16.67%) reflected on previous research read during the course and how the research could or could not be applied locally to the Macau context.

#### Research Engagement

Beliefs about research engagement are teachers' beliefs about “the degree of support for engaging with published research they felt was provided by their immediate school as well as their district” (Joram et al., 2020, p. 2). For example, participant #17 believed that “According to the class readings, games should play a central role in lessons and make it possible for children to fully immerse themselves in learning.” None of the participants mentioned such beliefs at the beginning or mid-way through the course; however, one participant (1.67%) did at the end of the course.

#### Microteaching

Teachers' beliefs about microteaching refer to teachers' feelings about the “technique in teacher education that uses short, specific episodes of teaching, usually videotaped, for analysis and instruction” (Harris and Hodges, 1995, p. 154). Sixteen participants (26.67%) described their beliefs about microteaching and how it assisted with their building of teaching competencies such as resilience, confidence, lesson planning abilities, and professional development. While none of the participants reflected on their beliefs about microteaching at the beginning or end of the course, 16 participants (26.67%) did reflect on their beliefs about microteaching mid-way through the course, right after they had completed an in-class peer microteaching activity.

## Discussion

The present study revealed five major themes, derived from 15 subthemes, regarding the pre-service pre-primary teachers' beliefs about teaching EFL to young learners in Macau. Generally, many previously documented beliefs themes were identified (Calderhead, 1996; Borg, 2003, 2006; Zheng, 2009; Shieh and Reynolds, 2020). Importantly, the findings also showed several beliefs subthemes that were not previously documented in the literature by pre-service pre-primary teachers such as epistemological beliefs informed by educational literature, beliefs about generalizability/transferability of research findings, and beliefs about research engagement. More importantly, this study investigated whether and how the teachers' beliefs changed over the *English Language Activities* course at three different time points.

A major finding is that substantial changes were documented in the pre-service pre-primary teachers' beliefs after they completed the course. During this teacher training course, the participants were provided with sufficient opportunities to learn, trial, and feel competent in teaching. The majority of the participants' beliefs changed from general beliefs to more concrete beliefs regarding English teaching to very young learners. This finding is in congruence with prior studies in that teacher education is one of the main factors influencing teachers' beliefs about teaching English in ECE contexts (Bekleyen, 2011; Brown and Englehardt, 2016; Brown, 2017; Brown et al., 2021). This suggests that the exposure to teacher training education courses is crucial in driving pre-service pre-primary teachers' beliefs toward the success of English teaching within ECE contexts.

Throughout the course, the participants became more aware of their learning to teach English by actively engaging in reflective activities. For example, the participants were asked to take notes during class and reflect on these notes to articulate their beliefs about teaching EFL. Reflective thinking was a facilitator in prompting the changes of beliefs during the course because it provided the participants with various opportunities to think about how they actually learn to teach English and how a particular set of pedagogical knowledge is age-appropriate for teaching very young EFL learners. This finding echoes the view of Chan (2016) that teacher education programs help pre-service pre-primary teachers to “enhance the understanding, practice and reflection of beliefs” (p. 428). Thus, they can feel competent by reflecting and adjusting their beliefs by providing age-appropriate teaching to very young learners. A reasonable explanation would be that teacher education programs which allocate resources to facilitate the development of innovative behaviors are the ones that can influence teachers' beliefs (Thurlings et al., 2015). Reflections help teachers to develop various innovative behaviors such as collecting teaching strategies, building content knowledge, and analyzing practices. These innovative behaviors may trigger the change in teachers' beliefs.

### Beliefs About Learners and Learning

Generally, most of the participants changed their beliefs about EFL learners and learning as a result of their participation in the *English Language Activities* course. At the beginning of the course, the participants could only manage to express some ECE pedagogical knowledge, English content knowledge, and teaching approaches at a superficial level. For most of the participants, this is because their prior experiences as pre-service teachers and with teacher education had only focused on general ECE without any specifics regarding ECE English teaching in Macau. Although the participants had learned the pedagogical knowledge and concepts for teaching young learners from other courses in their bachelor's program, they still had uncertainty in ECE English teaching. The participants faced difficulties in internalizing and reflecting on these initial beliefs. This finding is in line with that in Chan's (2016) study in that teacher training courses were associated with student teachers' beliefs. Furthermore, like Chan (2016), we found that our participants' lack of subject-based pedagogical knowledge prior to the course yielded only superficial beliefs in the teaching of English to very young learners. After the course, the learners in our study were able to provide more concrete beliefs with clearer justifications for said beliefs.

These pre-service pre-primary teachers adjusted their initial beliefs gradually by internalizing and reflecting on their own learning progress throughout the course. Their beliefs became more concrete and specific regarding learners and learning. They took into account learner developmental level in discussing child-centered, age-appropriate learning. For example, participant #8 developed a concrete belief that the learning goals of young children should be listening and speaking rather than writing because most learners are not ready to learn writing before the age of six. The participants believed in the need for selecting and adapting learning goals to cater to their learners' needs and capabilities. This finding supports the view of Alexiou (2020) that pre-service teachers need to be well-trained in both ECE and EFL in order for pre-primary EFL education to be effective.

### Beliefs About Teaching

Another important finding is the pre-service teachers' changes in their beliefs about teaching EFL in ECE contexts. These changes were reflected in the pre-service teachers' beliefs about classroom practices, the perceived impact of goals, lesson planning, and assessment. Moreover, the degree of change increased over time. This suggests that the changes in pre-service teacher beliefs occurring during the *English Language Activities* course are important for the effectiveness of teaching.

The changes in the participants' beliefs point toward an overall growth of constructive beliefs in teaching EFL in ECE contexts. Most participants had developed more sophisticated beliefs during their microteaching and at the end of this course, adjusting their beliefs about teaching toward facilitating child-centered knowledge construction. As revealed in Minor's et al. (2002) survey, student teachers appeared to hold a mix of transmissive beliefs and constructive beliefs at the beginning of their professional training. Ng and Rao (2008) also indicated that in-service ECE teachers who completed sufficient teacher education were likely to adjust their teaching beliefs by enthused constructivism. Teachers are shown to be willing to adjust at least part of their belief system for achieving the goals of teaching after receiving a teacher education program (Tatto, 1998; Brownlee et al., 2001; Altan, 2012). This change in the student teachers' beliefs suggests that they became more capable of critical thinking and decision making for their teaching by actively participating in a series of teacher training activities (e.g., role-playing and microteaching).

According to Debreli (2012), the factors influencing the adjustments of beliefs include student teachers' reflections on teacher training courses. The participants in the present study changed their beliefs in the directions of constructivism which emphasizes games, stories, and teaching through knowledge co-construction. These constructive beliefs also reflect contemporary international views of EFL teaching in ECE as proposed in various empirical studies (e.g., Aslanabadi and Rasouli, 2013; Chou, 2014; Karasimos, 2021). The reflection reports by the end of the course confirmed that the games and stories were present, to a different extent, in participants' concrete beliefs about teaching in the goals of EFL teaching, lesson planning, classroom practices, and assessments.

The concrete beliefs by the end of the course are more constructive than the beliefs at the beginning of the course. There are two possible reasons for this. First, the pre-service pre-primary teachers were influenced by their Macau local cultural values and their own experiences of teaching English (e.g., part-time English tutor experiences). Indeed, the pre-service teachers' own English learning styles and prior experiences had affected their beliefs. When they would have been in kindergarten, it was common for their teachers to assess their English ability in a formal manner and encourage them to aim for high marks on tests; the participants' own kindergarten English teachers emphasized repetition, recitation, and rote writing. Even though some of their previous kindergarten teachers used a variety of activities for teaching English, English academic achievement measured by traditional tests were still emphasized. Second, this course implemented teacher education successfully by providing the participants with sufficient opportunities to internalize and reflect on what they had learnt from the course. The adjustment and reformation of beliefs take time, and this was further enhanced by the period of microteaching. The microteaching period (i.e., mid-way through the course) was used as a transitional period for the participants to adjust their initial beliefs and to install new beliefs if necessary. Therefore, the design of microteaching may minimize the possibilities of dissonance between their beliefs and practice. The reflection reports mid-way through the course revealed that participants started to reflect on their performance in the microteaching; they had already entered a transition period in their reflections, and they had been in the process of accommodating to these changes. Therefore, this microteaching phase seamlessly drew the pre-service pre-primary teachers' attention to the change of beliefs.

### Beliefs About Subject

The findings revealed that the pre-service pre-primary teachers completed the *English Language Activities* course with various beliefs about content and pedagogies. These beliefs were mostly noted to be similar to the contemporary ECE language teaching trends of the subject knowledge and the pedagogical knowledge, which also aligns with contemporary teacher education courses.

One interesting finding is that instead of considering English as a kindergarten subject, some participants considered English as a tool of communication. This kind of belief reflected the participants' new ways of thinking about the status of English in ECE classes as they engaged in the *English Language Activities* course. An explanation for the causal origin of these new beliefs is that the participants had some personal experiences, received teacher education courses, spent some time thinking about the interdisciplinary issues in ECE and EFL, and ultimately arrived at their current beliefs. These beliefs were found in Fukuda's (2020) study with university EFL learners, but to the best of our knowledge, our study was the first one in which pre-service pre-primary English teachers have reported such beliefs.

The participants in the present study held some beliefs about pedagogical knowledge based on their previous learning experiences from other teacher education courses. Most of the participants' beliefs about this subtheme had been adjusted to cater to the content knowledge of EFL after they had completed the course. This is in line with the findings in previous studies in that content knowledge can facilitate a change in teachers' beliefs about pedagogical knowledge (e.g., Sung and Yang, 2013).

In addition, this study explored pre-service pre-primary teachers' beliefs about pedagogical knowledge more thoroughly and qualitatively by looking at various pedagogies embedded in the participants' beliefs. We found that the changes in beliefs regarding pedagogical knowledge happened to all of the participants; this goes in contrast to Paakkari's et al. (2015) study, which found only a few of their 20 participants adjusted their beliefs about pedagogical knowledge in health education.

One explanation for the pre-service teachers' changes in their beliefs is related to their age. For this age group of pre-service pre-primary teachers (20–21 years old), their beliefs were influenced by their teacher education courses (Tatto, 1998). The subject content knowledge of the courses (i.e., both ECE and EFL) may also have influenced their beliefs (Chong et al., 2005). Previous literature suggests that changes in beliefs about pedagogical knowledge are much easier at a younger age (Sheridan, 2016). Other studies support the rationale that beliefs about pedagogical knowledge are built within teacher education programs (Sperandeo-Mineo et al., 2006) and shaped by prior personal experiences as language learners (Stofflett and Stoddart, 1994) and language teachers (e.g., microteaching, practicum) (Mahmud and Rawshon, 2013). These studies confirm that there were differences in the degree of change in beliefs based on the pre-service pre-primary teachers' age.

### Beliefs About Self

The course resulted in some changes in the pre-service teachers' beliefs about self, including the role of teaching, hearsay, and self-evaluation. Their beliefs about the role of teaching clearly reflected “what teachers do in classrooms” after participating in the course (Keiler, 2018, p. 3). The pre-service teachers appeared to install new beliefs about the role of teaching to a higher level, which was reflected in their description of the coping strategies and responsibilities over the course. They considered that their views on the role of teaching ECE English influenced their classroom decision-making. The reported differences in beliefs at the three different stages of reflections suggest that the pre-service pre-primary English teachers judged their initial beliefs about the role of teaching and updated these beliefs to stronger beliefs by reflecting on what they had done in their microteaching sessions. Nafissi and Shafiee (2020) also suggest that central to the change is teacher education which focuses on nurturing ECE teachers' beliefs, awareness, and behaviors regarding their local ECE context.

The second change in the student teachers' beliefs about self-concerned their own schooling experiences. These pre-service teachers reported different beliefs about their critical moments in their learning journey. Background factors, their own English learning incidents, and past professional learning incidents seemed to shape their beliefs. In addition, as previous studies have shown, some beliefs about schooling can also be detrimental to constructive teaching in ECE (Duffy and Jonassen, 1991; Tomlinson, 1999; Hedges, 2000). Therefore, this finding can also suggest that teacher education courses can encourage student teachers to scaffold very young EFL learners' learning. This implies positive schooling experiences of the pre-service pre-primary English teachers aided in their processing the pedagogical and subject knowledge and thus further assisted them in reconceptualizing their beliefs about teaching EFL to pre-primary pupils.

### Beliefs About Learning to Teach

A final major finding of the study was some evidence of change in the pre-service teachers' journey of learning to teach EFL to young learners throughout the *English Language Activities* course. By the end of the course, the participants believed that high expectation pre-service teachers need to learn how to construct an appropriate teaching philosophy, how to choose suitable teaching methods, and how to develop age-appropriate assessments. This finding is consistent with that in a previous study conducted with Spanish EFL pre-service teachers (Agudo, 2014) in that the pre-service pre-primary teachers' beliefs about learning to teach were mainly influenced by their learning experiences. Bearing the results in mind, it can be concluded that a curriculum aimed at facilitating student teachers to become actively involved in their professional development require more hands-on activities such as microteaching and role-playing in their teacher education courses.

The pre-service teachers underwent a complex process of professional development throughout the *English Language Activities* course. They installed new beliefs such as their epistemological beliefs informed by educational literature, beliefs about generalizability/transferability of research findings, beliefs about research engagement, and beliefs about microteaching. This may be due to the influence of the teacher trainer who highlighted various issues and trends in the contemporary ECE and EFL education domains. Our participants had to think about what the related literature had to offer. Thus, the pre-service teachers were encouraged to search for answers from the research findings. In this way, the participants could learn to teach by synthesizing information from books, research journals, and the course readings. Furthermore, in group discussions, the participants worked with their peers to improve their professional skills. During the microteaching, the teacher trainer raised questions about their hands-on skills which further triggered their transferability of research findings.

## Limitations

In addition to identifying five major themes supported in the extant teacher beliefs literature, this case study also uncovered subthemes yet to be documented. While insightful, this case study is not limitation free. As with all case studies, the results cannot necessarily be generalized to a wider population. Instead, the results offer up contextualized findings applicable to one particular pre-primary teacher education curriculum in the Macau SAR context. Additional case studies in other institutions in the Macau SAR, Greater China, and the Asian EFL context are needed to gather further evidence for generalization to a wider population. Another option would be to supplement results from case studies such as this one with more wide-scale questionnaire studies executed under a quantitative research paradigm. In addition, the data collected for this study was limited to written reflections that only gathered the beliefs participants willingly reflected on. The participants could have held other beliefs that were not expressed in their written reflections. While it could be argued that the reflection on unprompted beliefs could be a sign that these were salient beliefs held by the participants, this cannot be confirmed without data triangulation. Future qualitative researchers working with a smaller data set may consider collecting interview data and pre-service teacher observation data to triangulate findings obtained from written reflections.

## Conclusion and Implications

The current study investigated the impact of an *English Language Activities* course on the pre-service teachers' beliefs about teaching English to preschool learners in Macau. The study adopted a qualitative case approach to explore the changes in the pre-service teachers' beliefs about various aspects of teaching EFL to young learners over the course. Analysis of the written reflections collected at three different stages throughout the course showed some important changes in the teachers' beliefs about various aspects of language teaching and learning.

The findings of the study identified five major themes and 15 subthemes related to pre-service teachers' beliefs about teaching EFL to young learners. These five themes, namely, (1) learners and learning, (2) teaching, (3) subject, (4) self, and (5) learning to teach, have been reported in previous literature (Calderhead, 1996; Borg, 2003, 2006). What is more notable is that the findings revealed several subthemes that have not been identified in the literature on pre-service teachers' beliefs. These subthemes included epistemological beliefs, beliefs about transferability of research findings, and beliefs about research engagement. These beliefs were only formed by the end of the course after the student teachers had done all the theoretical readings, classroom discussions, microteaching, and reflective activities.

Apart from showing some newly formed beliefs over the course, the findings also revealed some noticeable changes in the pre-service teachers' existing beliefs. Specifically, over the course, the teachers' beliefs became more specific and concrete. They took into account more learner and contextual factors when discussing aspects of effective teaching and learning for young learners. They referred particularly to their future students' developmental level and age-appropriate criteria in their discussions about teaching methodologies, designing and adapting learning activities and materials, and assessments.

The current study has several implications for teacher education courses. As revealed in the findings of the current study, the pre-service teachers displayed significant changes in their beliefs thanks to the exposure to the content of the course and the hands-on and reflective activities. Therefore, similar courses should provide students with sufficient theoretical knowledge with updated literature about language learning theories, learners, learning, and lesson planning, among others. Extensive discussion about these theoretical aspects can help student teachers shape or reshape their epistemological beliefs, beliefs about generalizability/transferability of research findings, and beliefs about research engagement. These beliefs are able to promote the formation or reformation of concrete beliefs about specific aspects of language teaching and learning. Secondly, it seems that hands-on and reflective activities helped the student teachers change their beliefs substantially. Most pre-service teachers in the present study shifted their beliefs from general to specific and concrete beliefs about learners, learning, subject matter, self, and learning to teach. Future teacher education courses may need to equip student teachers with sufficient hands-on and reflective activities to provide them with opportunities to reflect on, analyze, trial, and figure out what works for them and for their future students. In this way, they will be likely to develop meaningful knowledge and beliefs about various aspects of EFL teaching. Thirdly, hands-on and reflective activities should focus on the learner developmental and age-appropriate, context, and culture-specific teaching activities so that student teachers can well prepare for their subsequent practicum and teaching. Finally, teacher education courses may need to incorporate into the assessment some components of evaluation and feedback on context-specific teaching lessons. In this way, student teachers have opportunities to evaluate and learn to give constructive feedback on the demo-lessons. This process can help them reflect on the content and the experiential learning activities they attend throughout the course. As a sequence, the knowledge and beliefs that they develop over the course will be elaborated on and consolidated.

## Data Availability Statement

The raw data supporting the conclusions of this article will be made available by the authors, without undue reservation.

## Ethics Statement

The studies involving human participants were reviewed and approved by Research Services and Knowledge Transfer Office, University of Macau. The patients/participants provided their written informed consent to participate in this study.

## Author Contributions

BR and CD contributed to the conception and design of the study. BR secured funding for the study. SL organized the database. BR and SL performed the analysis that was further checked by XZ and CD. BR, SL, XH, and XZ drafted sections of the manuscript. BR and XH rewrote and revised the manuscript. All authors read and approved the submitted version.

## Conflict of Interest

The authors declare that the research was conducted in the absence of any commercial or financial relationships that could be construed as a potential conflict of interest.

## Publisher's Note

All claims expressed in this article are solely those of the authors and do not necessarily represent those of their affiliated organizations, or those of the publisher, the editors and the reviewers. Any product that may be evaluated in this article, or claim that may be made by its manufacturer, is not guaranteed or endorsed by the publisher.

## Appendix A

### Reflection Prompt

Write a reflection report concerning the teaching of English as a foreign language to very young language learners (i.e., kindergarteners). Your reflection should include your conception of teaching and learning English as a foreign language at the pre-primary level, a description of how you would teach English as a foreign language at the pre-primary level, and justification for why you would teach English as a foreign language in that way.

## References

[B1] Agudo J. M. (2014). Beliefs in learning to teach: EFL student teachers' beliefs about corrective feedback, in English as a Foreign Language Teacher Education, eds Agudo J. M. (Utrecht: Brill), 209–230.

[B2] Akaba S. Peters L. E. Liang E. Graves S. B. (2020). Pre-K teachers' perspectives on factors that influence their experiences through universal Pre-K policy changes in New York City. J. Early Child. Res. 18, 143–158. 10.1177/1476718X19885993

[B3] Aksoy E. (2017). Turkish student teachers' attitudes toward teaching in university-based and alternative certification programs in Turkey. Asia Pacific Educ. Rev. 18, 335–346. 10.1007/s12564-017-9475-8

[B4] Alacam N. Olgan R. (2019). Pre-service early childhood teachers' beliefs concerning parent involvement: the predictive impact of their general self-efficacy beliefs and perceived barriers. Education 47, 555–569. 10.1080/03004279.2018.1508244

[B5] Albaiz N. E. A. Ernest J. M. (2021). Exploring Saudi Kindergarten teachers' views and uses of school, family, and community partnerships practices. Early Childhood Educ. 49, 451–462. 10.1007/s10643-020-01091-z

[B6] Alexiou T. (2020). Introducing EFL in preschools: facts and fictions, in Advancing English Language Education, eds Zoghbor W Alexiou T. (Dubai: Zayed University Press), 61–74.

[B7] Altan M. Z. (2012). Pre-service EFL teachers' beliefs about foreign language learning. Eur. J. Teach. Educ. 35, 481–493. 10.1080/02619768.2011.643399

[B8] Aslanabadi H. Rasouli G. (2013). The effect of games on improvement of Iranian EFL vocabulary knowledge in kindergartens. Int. Rev. Soc. Sci. Human. 6, 186–195.

[B9] Atabek O. Burak S. (2020). Pre-school and primary school pre-service teachers' attitudes towards using technology in music education. Eurasian J. Educ. Res. 87, 47–68. 10.14689/ejer.2020.87.3

[B10] Ball D. L. Thames M. H. Phelps G. (2008). Content knowledge for teaching: what makes it special. J. Teach. Educ. 59, 389–407. 10.1177/0022487108324554

[B11] Bekleyen N. (2011). Can I teach English to children? Turkish preservice teacher candidates and very young learners. J. Early Child. Teach. Educ. 32, 256–265. 10.1080/10901027.2011.594700

[B12] Borg S. (2003). Teacher cognition in language teaching: a review of research on what language teachers think, know, believe, and do. Lang. Teach. 36, 81–109. 10.1017/S0261444803001903

[B13] Borg S. (2006). The distinctive characteristics of foreign language teachers. Lang. Teach Res. 10, 3–31. 10.1191/1362168806lr182oa

[B14] Borg S. (2011). The impact of in-service teacher education on language teachers' beliefs. System 39, 370–380. 10.1016/j.system.2011.07.009

[B15] Brown B. (2016). A systems thinking perspective on change processes in a teacher professional development programme. J. Educ. 66, 37–64. 10.17159/2520-9868/i66a02

[B16] Brown C. P. Englehardt J. (2016). Conceptions of and early childhood educators' experiences in early childhood professional development programs: a qualitative metasynthesis. J. Early Childhood Teacher Educ. 37, 216–244. 10.1080/10901027.2016.1204574

[B17] Brown C. P. Ku D. H. Barry D. P. (2021). Making sense of instruction within the changed kindergarten: perspectives from preservice early childhood educators and teacher educators. J. Early Child. Teach. Educ. 42, 20–52. 10.1080/10901027.2020.1726532

[B18] Brown J. P. (2017). Teachers' perspectives of changes in their practice during a technology in mathematics education research project. Teach. Teach. Educ. 64, 52–65. 10.1016/j.tate.2017.01.022

[B19] Brownlee J. Purdie N. Boulton-Lewis G. (2001). Changing epistemological beliefs in pre-service teacher education students. Teach. High. Educ. 6, 247–268. 10.1080/13562510120045221

[B20] Cabaroglu N. Roberts J. (2000). Development in student teachers' pre-existing beliefs during a 1-year PGCE programme. System 28, 387–402. 10.1016/S0346-251X(00)00019-1

[B21] Calderhead J. (1991). Images of teaching: student teachers' early conceptions of classroom practice. Teach. Teach. Educ. 7, 1–8. 10.1016/0742-051X(91)90053-R

[B22] Calderhead J. (1996). Teachers: beliefs and knowledge, in Handbook of Educational Psychology, eds Berliner D. C. Calfee R. C. (New York, NY: Macmillan), 709–725.

[B23] Chan W. L. (2016). The discrepancy between teachers' beliefs and practices: a study of kindergarten teachers in Hong Kong. Teach. Dev. 20, 417–433. 10.1080/13664530.2016.1161658

[B24] Cheung A. Hennebry-Leung M. (2020). Exploring an ESL teachers' beliefs and practices of teaching literary texts: a case study in Hong Kong. Lang. Teach. Res. 1–26. 10.1177/1362168820933447

[B25] Chong S. N. Y. Wong I. Y. F. Quek C. L. (2005). Pre-service teachers' beliefs, attitudes and expectations: a review of the literature. Paper Presented at the Redesigning Pedagogy: Research, Policy, Practice Conference (Singapore).

[B26] Chou M.-H. (2014). Assessing English vocabulary and enhancing young English as a foreign language (EFL) learners' motivation through games, songs, and stories. Education 42, 284–297. 10.1080/03004279.2012.680899

[B27] Clark-Goff K. Eslami Z. (2016). Exploring change in preservice teachers' beliefs about English language learning and teaching. Iranian J. Lang. Teach. Res. 4, 21–36. 10.30466/IJLTR.2016.20352

[B28] Cook V. (2013). What are the goals of language teaching? Iran. J. Lang. Teach. Res. 1, 44–56. 10.4304/jltr.2.4.929-933

[B29] Debreli E. (2012). Change in beliefs of pre-service teachers about teaching and learning English as a foreign language throughout an undergraduate pre-service teacher training program. Proc. Soc. Behav. Sci. 46, 367–373. 10.1016/j.sbspro.2012.05.124

[B30] Duffy T. M. Jonassen D. H. (1991). Constructivism: new implications for instructional technology? Educ. Technol. 31, 7–12.

[B31] Falknor J. F. (1940). Silence as hearsay. Univ. Pennsylvania Law Rev. Am. Law Regist. 89, 192–217. 10.2307/3308885

[B32] Farquhar S. White E. J. (2014). Philosophy and pedagogy of early childhood. Educ. Philos. Theor. 46, 821–832. 10.1080/00131857.2013.783964

[B33] Fukuda A. (2020). Exploring learner beliefs in self-regulated learning: a case investigation of an English self-study. JACET Select. Pap. 7, 91–120.

[B34] Gilakjani A. P. Sabouri N. B. (2017). Teachers' beliefs in English language teaching and learning: a review of the literature. English Lang. Teach. 10, 78–86. 10.5539/elt.v10n4p78

[B35] Ha X. V. Murray J. C. (2020). Corrective feedback: beliefs and practices of Vietnamese primary EFL teachers. Lang. Teach. Res. 1–31. 10.1177/1362168820931897

[B36] Ha X. V. Murray J. C. (2021). The impact of a professional development program on EFL teachers' beliefs about corrective feedback. System 96, 102–405. 10.1016/j.system.2020.102405

[B37] Ha X. V. Nguyen L. T. (2021). Targets and sources of oral corrective feedback in English as a foreign language classrooms: are students' and teachers' beliefs aligned? Front. Psychol. 12:697160. 10.3389/fpsyg.2021.697160 34248800 PMC8270110

[B38] Harris T. L. Hodges R. E. (1995). The Literacy Dictionary: The Vocabulary of Reading and Writing. International Reading Association.

[B39] Hedges H. (2000). Teaching in early childhood: time to merge constructivist views so learning through play equals teaching through play. Australas. J. Early Child. 25, 16–21. 10.1177/183693910002500404

[B40] Hiebert J. Morris A. K. Glass B. (2003). Learning to learn to teach: An “experiment” model for teaching and teacher preparation in mathematics. J. Math. Teach. Educ. 6, 201–222. 10.1023/A:1025162108648

[B41] Hood M. (2009). Case study, in Qualitative Research in Applied Linguistics: A Practical Introduction, eds Heigham J Croker R. A. (Basingstoke: Palgrave MacMillan), 66–90.

[B42] Hughes A. (2013). Testing for Language Teachers. Cambridge: Cambridge University Press.

[B43] Johnson K. E. (1994). The emerging beliefs and instructional practices of pre-service English as a second language teachers. Teach. Teach. Educ. 10, 439–452. 10.1016/0742-051X(94)90024-8

[B44] Joram E. Gabriele A. J. Walton K. (2020). What influences teachers' “buy-in” of research? Teachers' beliefs about the applicability of educational research to their practice. Teach. Teach. Educ. 88, 1–12. 10.1016/j.tate.2019.102980

[B45] Karasimos A. (2021). LetMeepleTalk: using board games for EFL preschoolers. Res. Pap. Lang. Teach. Learn. 11, 93–103.

[B46] Keiler L. S. (2018). Teachers' roles and identities in student-centered classrooms. Int. J. STEM Educ. 5:120. 10.1186/s40594-018-0131-6 30631724 PMC6310426

[B47] Korthagen F. A. J. Loughran J. Russell T. (2006). Developing fundamental principles for teacher education programs and practices. Teach. Teach. Educ. 22, 1020–1041. 10.1016/j.tate.2006.04.022

[B48] Kroll L. R. Laboskey V. K. (1996). Practicing what we preach: Constructivism in a teacher education program. Action Teach. Educ. 18, 63–72. 10.1080/01626620.1996.10462834

[B49] Leavy P. (Ed.). (2014). The Oxford Handbook of Qualitative Research. Oxford: Oxford University Press.

[B50] Levin B. B. (2015). The development of teachers' beliefs, in International Handbook of Research on Teachers' Beliefs, eds Fives H Gill M. G. (New York, NY: Routledge), 48–65.

[B51] Li Y. Oliveira H. (2015). Research on classroom practice, in The Proceedings of the 12th International Congress on Mathematical Education. 10.1007/978-3-319-12688-3_46

[B52] Lin Z. J. T. J. (2013). Language teachers' attitudes, beliefs, professional knowledge, and views on professional development: an exploratory study at a preschool TEFL setting. TESOL J. 4, 55–82. 10.1002/tesj.52

[B53] MacDonald M. Badger R. White G. (2001). Changing values: what use are theories of language learning and teaching? Teach. Teach. Educ. 17, 949–963. 10.1016/S0742-051X(01)00042-7

[B54] Mahmud I. Rawshon S. (2013). Micro teaching to improve teaching method: an analysis on students' perspectives. IOSR J. Res. Method Educ. 1, 69–76. 10.9790/7388-0146976

[B55] McLean M. Murdoch-Eaton D. Shaban S. (2013). Poor English language proficiency hinders generic skills development: a qualitative study of the perspectives of first-year medical students. J. Further High. Educ. 37, 462–481. 10.1080/0309877X.2011.645461

[B56] Miles M. B. Huberman A. M. (1994). Qualitative Data Analysis: An Expanded Sourcebook. Sage.

[B57] Minor L. C. Onwuegbuzie A. J. Witcher A. E. James T. L. (2002). Preservice teachers' educational beliefs and their perceptions of characteristics of effective teachers. J. Educ. Res. 96, 116–127. 10.1080/00220670209598798

[B58] Munez D. Bautista A. Khiu E. Keh J. S. Bull R. (2017). Preschool teachers' engagement in professional development: frequency, perceived usefulness, and relationship with self-efficacy beliefs. Psychol. Soc. Educ. 9, 181–199. 10.25115/psye.v9i2.655

[B59] Nafissi Z. Shafiee Z. (2020). Teachers' roles in early childhood English language pedagogy: beliefs of kindergarten English language teachers. J. Early Childhood Teach. Educ. 41, 306–324. 10.1080/10901027.2019.1647479

[B60] Ng S. S. Rao N. (2008). Mathematics teaching during the early years in Hong Kong: a reflection of constructivism with Chinese characteristics? Early Years 28, 159–172. 10.1080/09575140802020917

[B61] Paakkari L. Tynjälä P. Torppa M. Villberg J. Kannas L. (2015). The development and alignment of pedagogical conceptions of health education. Teach. Teach. Educ. 49, 11–21. 10.1016/j.tate.2015.02.005

[B62] Peacock M. (2001). Pre-service ESL teachers' beliefs about second language learning: a longitudinal study. System 29, 177–195. 10.1016/S0346-251X(01)00010-0

[B63] Reilly V. Ward S. M. (1997). Very Young Learners. Oxford: Oxford University Press.

[B64] Rolheiser C. Ross J. A. (2001). Student self-evaluation: what research says and what practice shows, in Plain Talk about Kids, eds Small R. D Thomas A. (Covington, LA: Center for Development and Learning), 43–57.

[B65] Schmidt M. (2010). Learning from teaching experience: Dewey's theory and preservice teachers' learning. J. Res. Music Educ. 58, 131–146. 10.1177/0022429410368723

[B66] Schreier M. (2012). Qualitative Content Analysis in Practice. London: Sage publications.

[B67] Sheridan L. D. (2016). Examining changes in pre-service teachers' beliefs of pedagogy. Australian J. Teach. Educ. 41, 1–20. 10.14221/ajte.2016v41n3.1

[B68] Shieh J.-J. Reynolds B. L. (2020). The origin and impact of an ESL teacher's beliefs on curriculum design. Asia Pacific J. Educ. 1–20. 10.1080/02188791.2020.1832043

[B69] Sinclair C. (2008). Initial and changing student teacher motivation and commitment to teaching. Asia Pacific J. Teach. Educ. 36, 79–104. 10.1080/13598660801971658

[B70] Sinclair C. Dowson M. McInerney D. (2006). Motivations to teach: psychometric perspectives across the first semester of teacher education. Teach. Coll. Rec. 108, 1132–1154. 10.1111/j.1467-9620.2006.00688.x

[B71] Siwatu K. (2007). Preservice teachers' culturally responsive teaching self-efficacy and outcome expectancy beliefs. Teach. Teach. Educ. 23, 1086–1101. 10.1016/j.tate.2006.07.011

[B72] Sperandeo-Mineo R. Fazio C. Tarantino G. (2006). Pedagogical content knowledge development and pre-service physics teacher education: a case study. Res. Sci. Educ. 36, 235–268. 10.1007/s11165-005-9004-3

[B73] Stofflett R. T. Stoddart T. (1994). The ability to understand and use conceptual change pedagogy as a function of prior content learning experience. J. Res. Sci. Teach. 31, 31–51. 10.1002/tea.3660310105

[B74] Sung P.-F. Yang M.-L. (2013). Exploring disciplinary background effect on social studies teachers' knowledge and pedagogy. J. Educ. Res. 106, 77–88. 10.1080/00220671.2012.658453

[B75] Tang S. Y. Cheng M. M. Cheng A. Y. (2014). Shifts in teaching motivation and sense of self-as-teacher in initial teacher education. Educ. Rev. 66, 465–481. 10.1080/00131911.2013.812061

[B76] Tatto M. T. (1998). The influence of teacher education on teachers' beliefs about purposes of education, roles, and practice. J. Teach. Educ. 49, 66–77. 10.1177/0022487198049001008

[B77] Teo T. Chai C. S. Hung D. Lee C. B. (2008). Beliefs about teaching and uses of technology among pre-service teachers. Asia Pac. J. Teach. Educ. 36, 163–174. 10.1080/13598660801971641

[B78] Thurlings M. Evers A. T. Vermeulen M. (2015). Toward a model of explaining teachers' innovative behavior: a literature review. Rev. Educ. Res. 85, 430–471. 10.3102/0034654314557949

[B79] Tomlinson P. (1999). Conscious reflection and implicit learning in teacher preparation. Part II: Implications for a balanced approach. Oxford Rev. Educ. 25, 533–544. 10.1080/030549899103973

[B80] Urmston A. (2003). Learning to teach English in Hong Kong: the opinions of teachers in training. Lang. Educ. 17, 112–137. 10.1080/09500780308666843

[B81] Vosniadou S. Lawson M. J. Wyra M. Van Deur P. Jeffries D. Ngurah D. I. G. (2020). Pre-service teachers' beliefs about learning and teaching and about the self-regulation of learning: a conceptual change perspective. Int. J. Educ. Res. 99, 1–17. 10.1016/j.ijer.2019.101495

[B82] Voss T. Kunter M. (2019). “Reality shock” of beginning teachers? Changes in teacher candidates' emotional exhaustion and constructivist-oriented beliefs. J. Teach. Educ. 71, 292–306. 10.1177/0022487119839700

[B83] Wall C. R. G. (2016). From student to teacher: changes in preservice teacher educational beliefs throughout the learning-to-teach journey. Teach. Dev. 20, 364–379. 10.1080/13664530.2016.1149509

[B84] Xu L. (2012). The Role of Teachers' beliefs in the language teaching-learning process. Theor. Pract. Lang. Stud. 2, 1397–1402. 10.4304/tpls.2.7.1397-1402

[B85] Yuan R. Mak P. Yang M. (2020). ‘We teach, we record, we edit, and we reflect': Engaging pre-service language teachers in video-based reflective practice. Lang. Teach. Res. 1–20. 10.1177/1362168820906281

[B86] Yuan R. Zhang L. J. (2017). Exploring student teachers' motivation change in initial teacher education: a Chinese perspective. Teach. Teach. Educ. 61, 142–152. 10.1016/j.tate.2016.10.010

[B87] Zheng H. (2009). A review of research on EFL pre-service teachers' beliefs and practices. J. Cambridge Stud. 4, 73–81. 10.17863/CAM.1579

[B88] Zimmerman B. J. (2008). Investigating self-regulation and motivation: historical background, methodological developments, and future prospects. Am. Educ. Res. J. 45, 166–183. 10.3102/0002831207312909

